# The oxygen uptake efficiency slope does not accurately predict $\dot{V}$ O_2peak_ of children – the Arkansas Active Kids study

**DOI:** 10.3389/fphys.2024.1358942

**Published:** 2024-09-26

**Authors:** Timothy Edwards, Elisabet Børsheim, Judith L. Weber, Eva C. Diaz

**Affiliations:** ^1^ Arkansas Children’s Nutrition Center, Little Rock, AR, United States; ^2^ Arkansas Children’s Research Institute, Little Rock, AR, United States; ^3^ Department of Pediatrics, University of Arkansas for Medical Sciences, Little Rock, AR, United States; ^4^ Department of Geriatrics, University of Arkansas for Medical Sciences, Little Rock, AR, United States

**Keywords:** oxygen uptake efficiency slope, peak VO_2_, children, body composition, bland altman

## Abstract

**Background:**

Cardiorespiratory fitness (CRF) is a vital indicator of health. However, accurately measuring peak oxygen consumption ( $\dot{V}$ O_2peak_) to determine CRF in children can be challenging. The oxygen uptake efficiency slope (OUES) has been proposed as an alternative metric for predicting $\dot{V}$ O_2peak_ in children, but its accuracy and agreement with measured $\dot{V}$ O_2peak_ remain unclear.

**Methods:**

A post hoc analysis was conducted in 94 children (ages 7–10 years) who completed an incremental cycle ergometer test to measure $\dot{V}$ O_2peak_. Body composition (Dual-energy X-ray absorptiometry) was measured, and fat mass index (FMI, kg/m^2^) and fat-free mass index (FFMI, kg/m^2^) were calculated. OUES was determined using all respiratory data (OUES_100%_) collected during the cycle ergometer test and using data only up to 60% of heart rate reserve (OUES_60%HRR_). Regression equations to predict $\dot{V}$ O_2peak_ (Pred- $\dot{V}$ O_2peak_) were derived from simple and multiple linear regression analysis. Bland-Altman analysis assessed the level of agreement between Pred- $\dot{V}$ O_2peak_ and measured $\dot{V}$ O_2peak_.

**Results:**

OUES_60%HRR_ (β = 0.46, *p* < 0.0001), age (β = 56.0, *p* = 0.0004), White race (β = 173.3, *p* < 0.0003), FFMI (β = 0.98.6, *p* < 0.000), and FMI (β = −0.40.8, *p* < 0.000) were retained in the final model. The difference between measured $\dot{V}$ O_2peak_ and Pred- $\dot{V}$ O_2peak_ was not different from zero (*p* = 0.999). There was a positive association between the difference of measured $\dot{V}$ O_2peak_ and Pred- $\dot{V}$ O_2peak_ and the average of the two methods (β = 0.79, *p* = 0.0028).

**Conclusion:**

There was no mean bias between measured $\dot{V}$ O_2peak_ and Pred- $\dot{V}$ O_2peak_. However, magnitude bias was present even after considering other significant predictors of $\dot{V}$ O_2peak_ (FMI, FFMI, race, and age) in the regression equation. Caution is advised when using OUES to predict $\dot{V}$ O_2peak_ in children.

## Introduction

Cardiorespiratory fitness (i.e., maximum oxygen consumption) is an independent predictor of all-cause mortality in adults, and an important marker of health in children (Imboden et al., 2018; Raghuveer et al., 2020). Cardiopulmonary exercise testing (CPET) is used to measure maximum oxygen consumption ( $\dot{V}$ O_2max_), the gold-standard measure for determining cardiorespiratory fitness, defined as a plateau in $\dot{V}$ O_2_ despite an increasing workload (Taylor et al., 1955). Measuring $\dot{V}$ O_2max_ in children can be challenging as only a fraction of them reach $\dot{V}$ O_2_ plateau (COOPER and WEILER-RAVELL, 1984), leading to the common utilization of $\dot{V}$ O_2peak_, which refers to the highest $\dot{V}$ O_2_ achieved during CPET. Most of the time, effort criteria such as the peak respiratory exchange ratio [carbon dioxide production ( $\dot{V}$ CO_2peak_)/ $\dot{V}$ O_2peak_], peak heart rate, and the rate of perceived exertion are used to assist in establishing $\dot{V}$ O_2peak_. However, factors such as obesity, underlying comorbidities, or insufficient personal motivation may impose limitations on children’s capacity to exert maximum effort and achieve the criteria for $\dot{V}$ O_2peak_. Consequently, this may result in the loss of data.

The oxygen uptake efficiency slope (OUES) is a submaximal metric utilized in CPET to assess the relationship between oxygen consumption ( $\dot{V}$ O_2_, mL/min) and minute ventilation ( $\dot{V}$ _E_, L/min) (Baba et al., 1996). OUES serves as a measure of ventilatory efficiency in relation to oxygen consumption during submaximal exercise. The OUES has found utility as an independent predictor of mortality in adult patients with idiopathic pulmonary hypertension and heart failure (Tang et al., 2017; Lin et al., 2016; Chiang et al., 2024). It is currently unknown whether the OUES can differentiate between healthy children and children with various diseases, but the evidence in adults suggests this.

More recently, the OUES was proposed as a predictor of $\dot{V}$ O_2peak_ in children (Akkerman et al., 2010; Dias et al., 2017; Breithaupt et al., 2012), but results are inconsistent across the literature (Drinkard et al., 2007; Sheridan et al., 2021). Two studies evaluated the comparability of measured $\dot{V}$ O_2peak_ and estimates of $\dot{V}$ O_2peak_ derived from the OUES (Pred- $\dot{V}$ O_2peak_) in adolescents with obesity (Sheridan et al., 2021), and in 15 years old male adolescents (Drinkard et al., 2007). The authors reported moderate to strong correlation between Pred- $\dot{V}$ O_2peak_ and measured $\dot{V}$ O_2peak_. However, the values derived from OUES showed large interindividual variation and magnitude bias suggesting that the OUES may not accurately predict $\dot{V}$ O_2peak_ in adolescents. The majority of studies concluding that the OUES is an acceptable alternative to $\dot{V}$ O_2peak_ have relied on correlation or regression statistics to compare both variables. A limitation of this approach is that correlations and regressions do not provide insight into bias or level of agreement between methods.

Studies evaluating the level of agreement between measured $\dot{V}$ O_2peak_ and Pred- $\dot{V}$ O_2peak_ in young children are lacking. To address this limitation, we conducted a secondary analysis examining the validity of OUES in estimating measured $\dot{V}$ O_2peak_ in 7-to-10-year-old children who participated in the Arkansas Active Kids study (NCT03221673). Based on the limited evidence in adolescents, we hypothesized that $\dot{V}$ O_2peak_ values predicted from OUES (Pred- $\dot{V}$ O_2peak_) do not consistently agree with individually measured $\dot{V}$ O_2peak_ across the range of $\dot{V}$ O_2peak_ values (from lower $\dot{V}$ O_2peak_ to higher $\dot{V}$ O_2peak_) in our cohort. Therefore, we hypothesized that OUES may not be a reliable predictor of $\dot{V}$ O_2peak_ in young children.

## Methods

### Subjects

This is a *post hoc* analysis of data collected from a subset of 94 children enrolled in the Arkansas Active Kids Study (AAK, NCT03221673) who met $\dot{V}$ O_2peak_ criteria as described in the *Measurements* section. The AAK study is a cross-sectional observational study aimed at identifying modifiable risk factors and phenotypes negatively associated with metabolic health of children. Details of the study protocol and design have been published elsewhere (Bai et al., 2020). Following an overnight fast, participants’ ages 7–10 years attended one study visit at the Arkansas Children’s Nutrition Center, Laboratory for Active Kids and Families. They were advised to avoid strenuous exercise the day before the study visit, but were not required to refrain from all physical activity. All participants were considered in good health. Exclusion criteria were type 1 or type 2 diabetes mellitus; severe persistent asthma (determined by daily use of oral/inhaled corticosteroid to keep asthma symptoms under control and/or frequent use of rescue inhaler) heart disease that required medication; neurological, kidney, liver, hormonal, lung or autoimmune disease; cancer; bleeding disorder; and pre-existing medical conditions or medications as determined by the investigators to affect the outcomes of interest. The Institutional Review Board at the University of Arkansas for Medical Sciences approved the study protocol. All parents and children gave written informed consent and assent, respectively.

## Measurements

### Anthropometry and body composition

Body weight (kg) and height (cm) were measured using a digital scale (Seca 877, Seca GbmH and Co. KG, Hamburg, Germany) to the nearest 0.1 kg and 0.1 cm, respectively, and triplicate values were averaged. Body mass index (BMI, kg/m^2^) was calculated and BMI percentiles were estimated using the Center for Disease Control and Prevention growth charts for ages 2–20 years (Canadian Pediatric Endocrine Group). Body composition was assessed using dual-energy X-ray absorptiometry (DXA, Horizon-A with Advanced Body Composition™, Hologic, Bedford, MA, United States). Fat mass (FM) index [FMI = FM (kg)/height^2^ (m^2^)] and fat-free mass (FFM) index [FFMI = FFM (kg)/height^2^ (m^2^)] were computed.

#### Cardiopulmonary exercise testing

$\dot{V}$ O_2peak_ was assessed through an incremental exercise test on a pediatric cycle ergometer (Corival Pediatric, Lode, Groningen, Netherlands). Seat height was modified using a goniometer to a corresponding knee angle of 150°. Incremental workload watts (W) were based on the participants’ height and were as follows: 10 W for children <125 cm tall, 15 W for children ≥125 < 150 cm tall, and 20 W for children ≥150 cm tall. Workloads increased in a stepwise fashion every minute throughout testing. Following a 1-min resting period and 1-min unloaded warmup, workload increased every minute, and participants were asked to keep a pedal frequency of 60–70 rpm. Participants were free to stop the test at any time but were encouraged to perform until voluntary exhaustion. Testing was terminated when the pedal frequency could not be maintained despite strong verbal encouragement. While testing, children were required to wear an indirect calorimetry facemask in order to assess their $\dot{V}$ O_2_, $\dot{V}$ CO_2_, and $\dot{V}$ _E_ (Medgraphics Ultima PFX^®^ system, MGC Diagnostics Corporation, St Paul, MN, United States). Prior to the tests, flow and gas calibrations were performed according to the manufacturer’s instructions. Participants also wore a heart rate monitor (Zephyr™ Heart Rate Monitor) or electrocardiogram (Mortara 12-Lead ECG) to measure heart rate during the exercise test. Participants initially underwent heart rate monitoring using Mortara 12-Lead ECG; however, to enhance practicality during the course of the study, we transitioned to the Zephyr™ Heart Rate Monitor.

#### Criteria for $\dot{V}$ O_2peak_

$\dot{V}$ O_2peak_ was determined using the highest 20-s average achieved during the final minute prior to exercise test termination. To classify the effort as a $\dot{V}$ O_2peak_, all three criteria had to be met: a rating of perceived exertion ≥8 on the OMNI scale (Utter et al., 2002), a peak heart rate ≥185 beats/min (or ≥85% of predicted peak heart rate), and a respiratory exchange ratio (RER) ≥ 1.0. Only children who met all three criteria were included in the analysis.

#### OUES determination

OUES is a slope calculation of $\dot{V}$ O_2_ *versus* logarithmically transformed $\dot{V}$ _E_ (in order to create a linear relationship) and is calculated based on this relationship during incremental exercise, where $\dot{V}$ _E_ (L/min) is positioned on the *x*-axis and $\dot{V}$ O_2_ (mL/min) on the *y*-axis. OUES is determined by equation $\dot{V}$ O_2_ = a log $\dot{V}$ _E_ + b, where ‘a' represents the OUES value (Baba et al., 1996). Higher $\dot{V}$ O_2_ values with lower $\dot{V}$ _E_ values create a steeper slope indicative of higher cardiorespiratory fitness (CRF) compared to lower slopes. The OUES slope including all respiratory data from the start of the workload up to test termination was assessed (OUES_100%_). If a plateau in $\dot{V}$ O_2_ occurred, subsequent data points were excluded. Additionally, to assess OUES at submaximal intensity, an OUES slope was calculated using data only from the start of the workload until a heart rate corresponding to 60% of heart rate reserve (HRR) was reached (OUES_60%HRR_). For the OUES_60%HRR_ method, HRR was first calculated by subtracting the participant’s resting heart rate from their age-predicted maximum heart rate (208–0.7 x age) (Tanaka et al., 2001). HRR was then multiplied by 0.6, and the participant’s resting heart rate added back to obtain the target heart rate. The slope was then calculated using data from the start of the workload until the target heart rate was reached.

### Statistical analysis

Data measures in the interval scale are summarized as mean ± SD whereas data measures in the ordinal or nominal scale are summarized as percentages and counts. Simple linear regression analysis modeled the association of measured $\dot{V}$ O_2peak_ (ml/min, dependent variable) with OUES_60%HRR_, age (years), sex, race (White and Black), BMI percentile, FMI, and FFMI (independent variables). For statistical analysis, sex and race were dichotomized as 0 or 1, with 0 representing girls and Black individuals, and 1 representing boys or White individuals, respectively. The best-fitted model was created using stepwise multiple linear regression analysis. Regression equations to predict $\dot{V}$ O_2peak_ were derived from simple and multiple linear regression analysis. Regression analyses are presented as parameter estimate (β), standard error (SE), 95% confidence interval (95% CI), R-squared (R^2^), and variance of inflation factor (VIF) when applicable.

Bland-Altman analysis (Bland and Altman, 1986) was used to evaluate the level of agreement between predicted values $\dot{V}$ O_2peak_ (Pred- $\dot{V}$ O_2peak_) obtained from regression analysis, and $\dot{V}$ O_2peak_ measured using gold standard techniques. Mean bias between methods was assessed using a two-sample t-test. Magnitude bias was assessed by regressing the difference between the methods on the average of the two methods. The Pearson correlation coefficient was used to measure the correlation of measured $\dot{V}$ O_2peak_ with OUES_60%HRR_, OUES_100%_, and Pred- $\dot{V}$ O_2peak._ Statistical analyses were conducted with SAS^®^ 9.4 (Cary, NC, U.S.A).

## Results

### Participant characteristics

Children (9.1 ± 1.2 years) were predominantly White (85%), with Black children comprising 15% of the cohort. The proportion of girls was higher compared to boys (62% vs. 38%, *p* = 0.0233). Thirty percent of children had overweight or obesity while 70% of children had normal weight (Table 1).

**TABLE 1:** Subject characteristics.

Variable	n = 94
Age (years)	9.08 ± 1.22
Sex, n (%)	
Boys	36 (38)*
Girls	58 (62)
Race, n (%)	
Black	14 (15)
White	80 (85)
Fat mass index, (kg/m^2^)	6.41 ± 2.43
Fat-free mass index, (kg/m^2^)	12.0 ± 1.44
BMI (kg/m^2^)	18.6 ± 3.5
BMI percentile	61.17 ± 27.90
BMI status, n (%)	
Normal weight	66 (70)
Overweight	16 (17)
Obesity	12 (13)
$\dot{V}$ O_2peak_ (mL/min)	1198.95 ± 270.73
$\dot{V}$ O_2peak_ (ml·kg^-1^·min^-1^)	36.64 ± 8.00
Peak heart rate (bpm)	187.53 ± 9.09
Peak respiratory exchange ratio	1.05 ± 0.04
Peak OMNI exertion rating	10.00 ± 0
Resting heart rate (bpm)	70.54 ± 9.93

Data presented as mean ± SD, counts, and percentages; BMI, body mass index.

^*^ The proportion of girls is significantly higher compared to boys (*p* = 0.0233).

### Bivariate associations between $\dot{V}$ O_2peak_ (mL/min) and variables of interest

$\dot{V}$ O_2peak_ was independently and positively associated with OUES_60%HRR_ (β = 0.85, *p* < 0.0001), age (β = 133.8, *p* < 0.0001), male sex (β = 184.3, *p* = 0.0011), BMI percentile (β = 2.0, *p* = 0.0448), and FFMI (98.4, *p* = 0.0019) (Table 2).

**TABLE 2:** Bivariate associations between V̇O_2peak_ (ml/min, dependent variable) with the oxygen uptake efficiency slope measured using data up to 60% of heart rate reserve (OUES_60%HRR_, mL/min) and other variables of interest.

Variables	β	SE	R^2^	95% CI		*p*-value
Age (years)	133.78	18.57	0.35	96.91	170.66	<0.0001
Sex, n (%)						
Boys	184.26	54.46	0.10	76.09	292.44	0.0011
Girls (reference)						
Race						
White	125.15	77.77	0.02	−29.31	279.61	0.1110
Black (reference)						
BMI percentile	2.01	0.99	0.03	0.05	3.98	0.0448
FMI (kg/m^2^)	4.37	11.60	0.01	−18.67	27.41	0.7072
FFMI (kg/m^2^)	98.40	30.80	0.23	57.55	125.45	0.0019
OUES_60%HRR_ (mL/min)	0.85	0.09	0.51	0.68	1.02	<0.0001

SE, standard error; CI, confidence Interval; BMI, body mass index; FMI, fat mass index; FFMI, fat-free mass index; OUES_60%HRR_, oxygen uptake efficiency slope measured using data up to 60% of heart rate reserve.

### Results from stepwise multiple linear regression analysis modeling the association of $\dot{V}$ O_2peak_ (mL/min) with subject characteristics and OUES_60%HRR_

Age, race, sex, FFMI, FMI, height, and OUES_60%HRR_ were all considered in the analysis. OUES_60%HRR_ (β = 0.46, *p* < 0.0001), age (β = 56.0, *p* = 0.0004), White race (β = 173.3, *p* < 0.0003), FFMI (β = 0.98 *p* < 0.000), and FMI (β = −0.40.8, *p* < 0.000) were retained in the final model. Sixty-nine percent of the observed variance in $\dot{V}$ O_2peak_ was predicted by this model (Table 3).

**TABLE 3:** Best fitted models from stepwise multiple linear regression analysis modeling the association of measured V̇O_2peak._ (dependent variable) with the oxygen uptake efficiency slope measured using data up to 60% of heart rate reserve (OUES_60%HRR_, mL/min), age, race, fat free mass index, and fat mass index (independent variables).

Variable	β	SE	Partial R^2^	VIF	95% CI			*p*-Value		Model Adjusted R2
OUES_60%HRR_ (mL/min)	0.46	0.09	0.24	1.67	0.29	0.64	<0.0001		0.69	
Age	55.95	15.09	0.14	1.39	25.97	85.93	0.0004			
Race										
White	173.29	46.56	0.14	1.14	80.76	265.82	0.0003			
Black (Reference)										
FFMI (kg/m^2^)	98.59	18.53	0.24	2.94	61.75	135.42	<0.0001			
FMI (kg/m^2^)	−40.79	9.44	0.17	2.17	−59.56	−22.02	<0.0001			

SE, standard error; CI, confidence Interval; VIF, variance of inflation factor; OUES60%HRR, oxygen uptake efficiency slope measured using data up to 60% of heart rate reserve; FFMI, fat-free mass index; FMI, fat mass index.

### Regression equations used to predict $\dot{V}$ O_2peak_ (mL/min) from OUES_60%HRR_ and other variables of interest

Equation 1 derived from the bivariate association between $\dot{V}$ O_2peak_ and OUES_60%HRR_

$$\dot{V}O_{2\text{peak}}\;\left(\text{ml}/\min\right)=192.07+0.8491\times {\text{OUES}}_{60\%\text{HRR}}$$ (1)

Equation 2 derived from multiple linear regression analysis in which OUES and other significant explanatory variables are considered.

$$\dot{V}O_{2\text{peak}}\;\left(\text{ml}/\min\right)=‐932.02+0.4618\times {\text{OUES}}_{60\%\text{HRR}}+55.95\times \text{age}+173.29\times \text{race}+98.585\times \text{LBMI }‐\;40.788\times \text{FMI}$$ (2)

### Correlation of Pred- $\dot{V}$ O_2peak_ (mL/min) with OUES_60%HRR,_ and OUES_100%_

**FIGURE 1:**
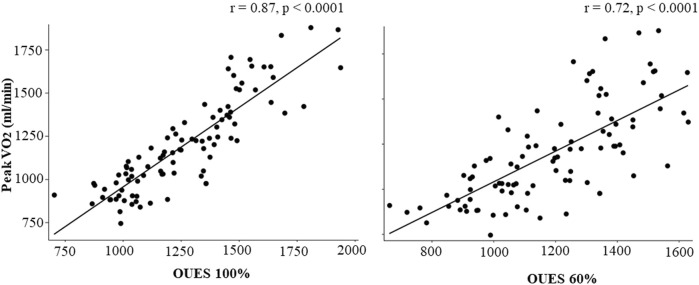
Scatter plots showing the correlation of the oxygen uptake efficiency slope (OUES) measured using all respiratory data (OUES_100%_) and using data up to 60% of heart rate reserve (OUES_60%HRR_).

**FIGURE 2:**
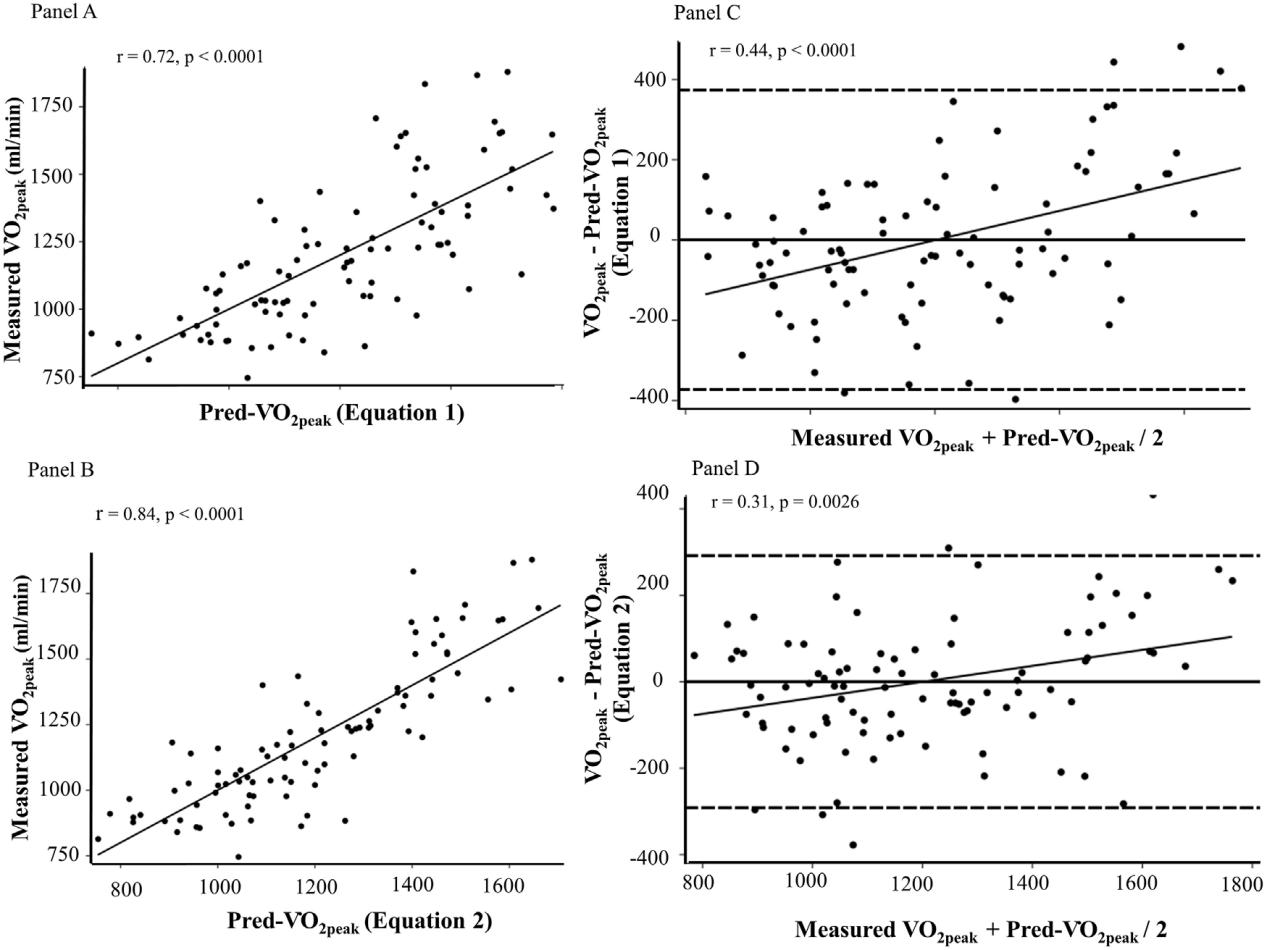
Scatter plots showing the correlation of Pred- $\dot{V}$ O_2peak_ (*x*-axis) estimated from Equation 1 (Panel A) and 2 (Panel B) with measured $\dot{V}$ O_2peak_ (*y*-axis). Bland-Altman plots constructed using measured $\dot{V}$ O_2peak_ and OUES_60%HRR_ from Equation 1 (Panel C) and 2 (Panel D). The difference between Pred- $\dot{V}$ O_2peak_ and OUES_60%HRR_ (*y*-axis) was plotted against the average of the two methods (*x*-axis). The dashed lines represent the two standard deviation limits.

OUES_60%HRR_ (r = 0.72, *p* < 0.0001) and OUES_100%_ (r = 0.87, *p* < 0.0001) positively correlated with measured $\dot{V}$ O_2peak_ (Figure 1). Pred- $\dot{V}$ O_2peak_ estimated from Equation 1 (r = 0.72, *p* < 0.0001) and Equation 2 (r = 0.84, *p* < 0.0001) positively correlated with measured $\dot{V}$ O_2peak_ (Figure 2, Panel A and B).

### Agreement between measured $\dot{V}$ O_2peak_ (mL/min) and Pred- $\dot{V}$ O_2peak_ (mL/min)

The average of Pred- $\dot{V}$ O_2peak_ obtained from Equation 1 (1198.95 ± 270.73 mL/min) and 2 (1198.95 ± 227.92 mL/min) were comparable to the average of measured $\dot{V}$ O_2peak_ (1198.95 ± 270.73, *p* = 0.9999). The Bland-Altman plots (Figure 2, Panel C and D) show the mean of the difference of measured $\dot{V}$ O_2peak_ - Pred- $\dot{V}$ O_2peak_ at zero (solid black line) thus indicating no mean bias between methods with neither Equation 1 (mean difference ± 2SD: 0.0 ± 372.9, p = NS) nor Equation 2 (mean difference = 0.0 ± 311.3). There was magnitude bias as indicated by the positive association between the difference of measured $\dot{V}$ O_2peak_–Pred- $\dot{V}$ O_2peak_ (*y*-axis) and the average of the two methods (*x*-axis) when Pred- $\dot{V}$ O_2peak_ estimates were computed using Equation 1 [β = 0.38 (confidence level (CL) = 0.22–0.55), *p* < 0.0001] and 2 [β = 0.79 (CL = 0.07–0.31), *p* = 0.0028].

## Discussion

The aim of this study was to evaluate the level of agreement between directly measured $\dot{V}$ O_2peak_ (mL/min) with $\dot{V}$ O_2peak_ estimated from OUES_60%HRR_ in school-age children. Our findings showed no mean bias between methods. However, magnitude bias was present even after considering other significant predictors of $\dot{V}$ O_2peak_ (FMI, FFMI, race, and age) in the regression equation. Specifically, there was an increase in bias (the difference between methods) as the magnitude of the measurements increased (the mean of both methods). The presence of magnitude bias indicates that the accuracy of the prediction varies depending on the level of $\dot{V}$ O_2peak_, which may limit the interchangeability of OUES-derived $\dot{V}$ O_2_peak predictions with directly measured $\dot{V}$ O_2peak_.

Contrary to previous studies that have supported the interchangeability of OUES and $\dot{V}$ O_2max_ or $\dot{V}$ O_2peak_ in school-age children, our results caution against this practice. We observed positive correlations between OUES_60%HRR_ and OUES_100%_ with measured $\dot{V}$ O_2peak_, as well as with predicted $\dot{V}$ O_2peak_ using two different equations. However, the Bland-Altman plots revealed magnitude bias (Figure 2, Panels C and D), indicating that the agreement between measured $\dot{V}$ O_2peak_ and OUES-predicted $\dot{V}$ O_2peak_ varies across the range of measurements. Consequently, we found large interindividual variations between the OUES-predicted $\dot{V}$ O_2peak_ and the measured $\dot{V}$ O_2peak_ despite identical group averages (1198.95 ± 270.73 mL/min and 1198.95 ± 270.73, respectively). These results underscore the importance of considering not only mean bias and correlation, but also magnitude bias when evaluating the agreement between methods. Submaximal moderate intensity estimate methods, like the OUES_60%HRR_, may be useful as a rough estimate of CRF in scenarios where maximal testing is impractical or not well-tolerated. However, as our findings demonstrate, while a submaximal OUES end-point provides a strong correlation, the presence of magnitude bias indicates that caution should be used when attempting to apply submaximal estimates interchangeably with directly measured $\dot{V}$ O_2peak_. Our findings highlight the importance of considering both the convenience of submaximal measures and their limitations in accurately representing CRF.

The concept of OUES was first introduced by Baba et al. (Baba et al., 1996) as an index of cardiorespiratory reserve derived from submaximal data during an incremental exercise test. The authors reported this index to be a useful indicator of cardiorespiratory efficiency in children (mean age 11.7 ± 4.4 years) that has a strong correlation to $\dot{V}$ O_2max_. Thus, they concluded it may be a helpful tool to aid clinicians in monitoring cardiorespiratory status over time. Following its introduction, several studies have evaluated the use of OUES as an indicator of cardiorespiratory fitness in a wide range of populations. There has been a consistently strong correlation seen between OUES and $\dot{V}$ O_2max_ or $\dot{V}$ O_2peak_ (Breithaupt et al., 2012; Drinkard et al., 2007; Sheridan et al., 2021; Pichon et al., 2002; Marinov et al., 2007) in all populations studied (healthy, overweight/obese, cardiovascular and pulmonary disease, etc.). Therefore, the majority of these studies typically report on its potential as a surrogate for $\dot{V}$ O_2max_ or $\dot{V}$ O_2peak_, especially in those who are unable to give maximum effort. However, Drinkard et al., 2007, Sheridan et al., 2021; Pichon et al., 2002 all noted the wide limits of agreement found by using Bland-Altman analysis, with Pichon observing a ±10.5 mL kg^-1^·min^-1^ limit of agreement. A strong limitation of the published evidence in children has been the generalized use of correlation coefficients and linear regression analysis as indicators of agreement between OUES-derived estimates of $\dot{V}$ O_2peak_ and measured $\dot{V}$ O_2peak_. Correlation is a metric that quantifies the degree of a linear relationship, rather than indicating agreement. For instance, a change in the scale of a measurement does not impact the correlation, but it does affect the agreement. Therefore, inferring that methods can be used interchangeably based solely on a high correlation is not recommended.

Marinov et al. (Marinov et al., 2007) studied 114 children (58 boys and 56 girls) ages 7–18 years to assess the correlation between OUES and $\dot{V}$ O_2peak_. The authors found no difference in OUES values estimated up to the anaerobic threshold (2600 ± 650 mL/min) and up to peak exercise (2600 ± 650 mL/min). In addition, there was a strong correlation between OUES and $\dot{V}$ O_2peak_ (r = 0.92). In light of this, the authors concluded that OUES is an objective measure of exercise capacity in the pediatric population. However, the study relied on correlation analysis to draw their conclusion, which does not provide a comprehensive assessment of agreement. Similarly, in our study, we observed strong correlations between OUES-derived predicted equations (Equation (1) r = 0.72; Equation (2) r = 0.84) with measured $\dot{V}$ O_2peak_. However, the absence of magnitude bias assessment in the Marinov et al. study limits the evaluation of OUES accuracy in predicting $\dot{V}$ O_2peak_. Consequently, our Bland-Altman analysis suggests differing conclusions despite similar methods and correlation strengths.

Only two studies in adolescents (Drinkard et al., 2007; Sheridan et al., 2021) have used alternative statistical approaches to evaluate the comparability between methods. Drinkard et al. (Drinkard et al., 2007) studied n = 150 (n = 107 with obesity and n = 43 with normal weight) adolescents. Initially, 141 adolescents with obesity were screened for eligibility, but 34 of them (24%) did not meet peak criteria and were not included in the analysis. This highlights the importance of exploring alternative methods for predicting $\dot{V}$ O_2peak_ in children with obesity. The authors evaluated the level of agreement in predicting $\dot{V}$ O_2peak_ using OUES at different exercise intensities. Magnitude bias was observed across all intensity levels with large limits of agreement (±478 to ±670 mL/min) despite a moderate to strong correlation range (r = 0.35–0.83). Consequently, the authors concluded that OUES-derived predictions of $\dot{V}$ O_2peak_ offer limited value in predicting CRF in individuals. Similarly, Sheridan et al. (Sheridan et al., 2021) reported magnitude bias with large limits of agreement (±937.5 mL/min) between OUES-predicted and measured $\dot{V}$ O_2peak_ of healthy 15-year-old males, despite a strong correlation (r = 0.77) between the two. Our findings align with these studies in adolescents, as we observed magnitude bias with large limits of agreement (±311.3 mL/min) despite strong correlation strength. Performing Bland-Altman analysis in our study provided a more comprehensive evaluation of OUES-derived predictions of $\dot{V}$ O_2peak_. While our best-fitted model yielded narrower limits of agreement than Drinkard et al., 2007; Sheridan et al., 2021, there is still significant magnitude bias, and a large variation up to ±311.3 mL/min. This highlights the need for a more accurate OUES-derived equation before interchangeable use with $\dot{V}$ O_2peak_. Therefore, our study helps fill the existing knowledge gap regarding the application of OUES as a means to predict peak cardiopulmonary capacity in children across developmental stages.

## Limitations

Participants in this study were predominantly White; therefore, extrapolation of these results to other races must be done with caution. While our criteria for classifying $\dot{V}$ O_2peak_ are in line with current pediatric recommendations, it should be acknowledged that this variable could be prone to underestimation in this population if using less conservative criteria.

One could argue a limitation of our study is the use of the same group of subjects for both deriving the OUES method and subsequently testing it against measured $\dot{V}$ O_2peak_. This methodological choice likely led to the zero mean bias observed in the Bland-Altman analysis, as the prediction equations were optimized based on the same dataset. This bias could give the impression that the OUES method is more accurate than it might be if applied to an independent cohort. It is important to recognize that using an independent group of subjects for validation is a more robust approach, as it reduces the risk of overfitting and provides a clearer picture of the method’s generalizability. In future studies, we recommend that the OUES method be validated in a separate cohort to ensure that the bias is not artificially minimized, and to better assess the true predictive performance of the method. Despite this limitation, the Bland-Altman analysis in our study still provides valuable information about the variability between the estimated and measured $\dot{V}$ O_2peak_ values. However, readers should interpret the zero mean bias with caution, understanding that it may not fully reflect the method’s accuracy in a broader population.

## Experimental considerations

The study is strengthened by the use of direct measurements of body composition and the inclusion of multiple variables in the regression analysis, which enhances the comprehensiveness of the study into factors influencing the use of OUES as a predictor of $\dot{V}$ O_2peak._ While our study emphasizes caution in interpreting the OUES as an accurate predictor for $\dot{V}$ O_2peak_ in children, the OUES still offers important insights into cardiorespiratory efficiency during submaximal exercise, especially in those who are unable or unwilling to give maximum effort. Clinicians and researchers should consider the context of its use, and the goal of their assessments when deciding to use the OUES. Future research should focus on developing novel models that better reflect the relationship between $\dot{V}$ O_2_ and $\dot{V}$ _E_ by incorporating additional variables or further adjustments that account for individual differences. Accurate estimates of $\dot{V}$ O_2peak_ in children with varying ranges of cardiorespiratory fitness and BMI are needed. OUES estimates of $\dot{V}$ O_2peak_ and gold standard measurements of $\dot{V}$ O_2peak_ do not agree equally throughout the range of measurements. Therefore, caution is advised when using OUES to predict $\dot{V}$ O_2peak_ in children.

In conclusion, our comprehensive OUES-derived equation, incorporating age, race, sex, FFMI, and FMI, demonstrated a strong correlation with measured $\dot{V}$ O_2peak._ However, the presence of magnitude bias and large interindividual variation indicates that the OUES is not an accurate predictor of CRF in children.

## Data Availability

The raw data supporting the conclusions of this article will be made available by the authors, without undue reservation.
